# 3-(*p*-Anis­yl)sydnone

**DOI:** 10.1107/S1600536810047422

**Published:** 2010-11-20

**Authors:** Hoong-Kun Fun, Jia Hao Goh, Nithinchandra, B. Kalluraya

**Affiliations:** a X-ray Crystallography Unit, School of Physics, Universiti Sains Malaysia, 11800 USM, Penang, Malaysia; b Department of Studies in Chemistry, Mangalore University, Mangalagangotri, Mangalore 574 199, India

## Abstract

In the title sydnone compound [systematic name: 3-(4-meth­oxy­phen­yl)-1,2,3-oxadiazol-3-ium-5-olate], C_9_H_8_N_2_O_3_, the essentially planar oxadiazole ring [maximum deviation = 0.005 (1) Å] is inclined at a dihedral angle of 30.32 (8)° with respect to the benzene ring. In the crystal, adjacent mol­ecules are inter­connected by inter­molecular C—H⋯O hydrogen bonds into sheets lying parallel to (100). Weak inter­molecular π–π inter­actions [centroid–centroid distance = 3.5812 (8) Å] further stabilize the crystal packing.

## Related literature

For general background to and applications of the title sydnone compound, see: Hegde *et al.* (2008 ▶); Kalluraya & Rahiman (1997 ▶); Kalluraya *et al.* (2002 ▶); Rai *et al.* (2008 ▶). For closely related sydnone structures, see: Goh *et al.* (2010*a* ▶,*b* ▶,*c* ▶). For the stability of the temperature controller used in the data collection, see: Cosier & Glazer (1986 ▶).
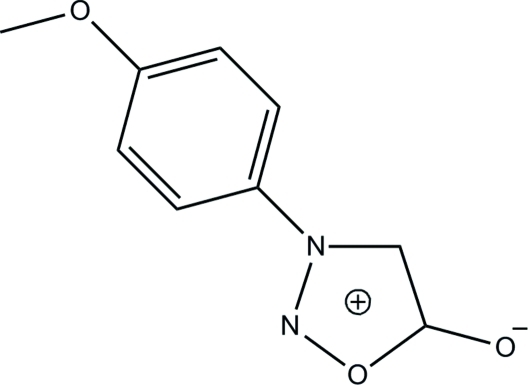


## Experimental

### 

#### Crystal data

C_9_H_8_N_2_O_3_
*M* *_r_* = 192.17
Orthorhombic, 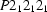

*a* = 7.0505 (2) Å
*b* = 9.8220 (3) Å
*c* = 12.0934 (3) Å
*V* = 837.47 (4) Å^3^
*Z* = 4
Mo *K*α radiation
μ = 0.12 mm^−1^
*T* = 100 K
0.56 × 0.15 × 0.14 mm

#### Data collection

Bruker SMART APEXII CCD area-detector diffractometer
Absorption correction: multi-scan (*SADABS*; Bruker, 2009 ▶) *T*_min_ = 0.938, *T*_max_ = 0.984
7384 measured reflections
1760 independent reflections
1577 reflections with *I* > 2σ(*I*)
*R*_int_ = 0.031

#### Refinement

*R*[*F* ^2^ > 2σ(*F* ^2^)] = 0.037
*wR*(*F* ^2^) = 0.100
*S* = 1.06
1760 reflections
159 parameters
All H-atom parameters refined
Δρ_max_ = 0.32 e Å^−3^
Δρ_min_ = −0.26 e Å^−3^

### 

Data collection: *APEX2* (Bruker, 2009 ▶); cell refinement: *SAINT* (Bruker, 2009 ▶); data reduction: *SAINT* (Bruker, 2009 ▶); program(s) used to solve structure: *SHELXTL* (Sheldrick, 2008 ▶); program(s) used to refine structure: *SHELXTL*; molecular graphics: *SHELXTL*; software used to prepare material for publication: *SHELXTL* and *PLATON* (Spek, 2009 ▶).

## Supplementary Material

Crystal structure: contains datablocks global, I. DOI: 10.1107/S1600536810047422/rz2524sup1.cif

Structure factors: contains datablocks I. DOI: 10.1107/S1600536810047422/rz2524Isup2.hkl

Additional supplementary materials:  crystallographic information; 3D view; checkCIF report

## Figures and Tables

**Table 1:** Hydrogen-bond geometry (Å, °)

*D*—H⋯*A*	*D*—H	H⋯*A*	*D*⋯*A*	*D*—H⋯*A*
C1—H1*A*⋯O2^i^	1.00 (2)	2.59 (2)	3.5441 (19)	159.1 (15)
C7—H7*A*⋯O3^ii^	0.98 (2)	2.42 (2)	3.3814 (18)	165.7 (17)

Symmetry codes: (i) 
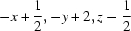
; (ii) 
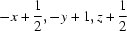
.

**Fig. 1.**
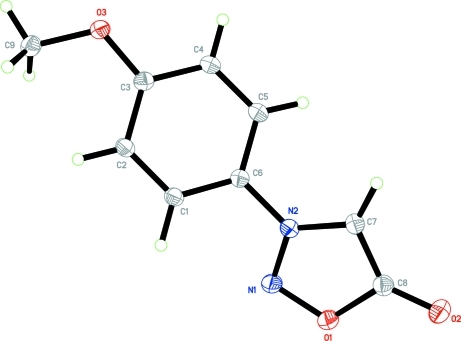
The molecular structure of the title sydnone compound, showing 50 % probability displacement ellipsoids for non-H atoms and the atom-numbering scheme.

**Fig. 2.**
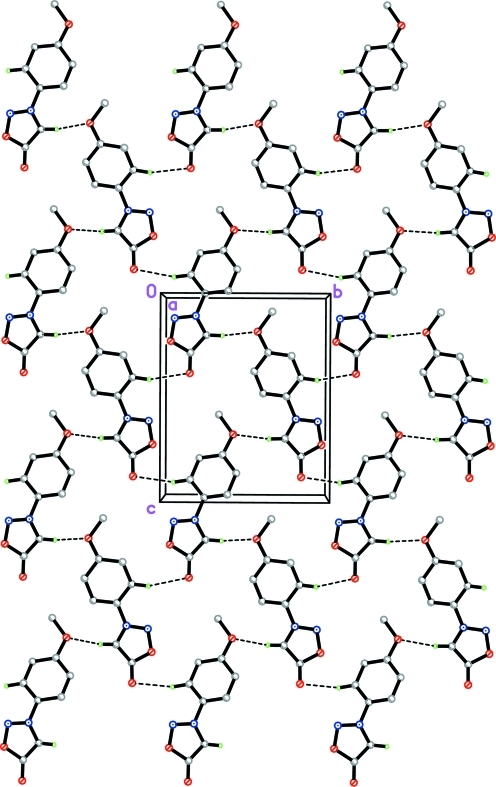
The crystal structure of the title compound, viewed along the a axis, showing a two-dimensional sheet parallel to the bc plane. H atoms not involved in intermolecular hydrogen bonds (dashed lines) have been omitted for clarity.

## supplementary crystallographic information

### Comment

Sydnones constitute a well-defined class of mesoionic compounds consisting of 1,2,3-oxadiazole ring system. The study of sydnones still remains a field of interest because of their electronic structure and also because of the varied types of biological activity displayed by some of them (Rai *et al.*, 2008). Sydnone derivatives were found to exhibit promising anti-microbial properties (Hegde *et al.*, 2008). Sydnones are synthesized by the cyclodehydration of *N*-nitroso-*N*-substituted amino acids using acetic anhydride. The sydnones unsubstituted in the 4-position readily undergo typical electrophilic substitution reaction namely formylation (Kalluraya & Rahiman, 1997) and acetylation (Kalluraya *et al.*, 2002).

In the title sydnone compound (Fig. 1), the 1,2,3-oxadiazole ring with atom sequence C7/C8/O1/N1/N2 is essentially planar, with a maximum deviation of 0.005 (1) Å at atom O1. The whole molecule is not planar, as indicated by the dihedral angle formed between the 1,2,3-oxadiazole and phenyl rings of 30.32 (8)°. Comparing with those previously reported structures with substitution at the 4-position of the sydnone moiety (Goh *et al.*, 2010*a*,*c*), the exocyclic C8—O2 bond length [1.2174 (8) Å] is longer than the respective values observed [1.193 (3) and 1.2089 (9) Å]. All other geometric parameters agree well with those observed in closely related sydnone structures (Goh *et al.*, 2010*a*,*b*,*c*).

In the crystal packing, intermolecular C1—H1A···O2 and C7—H7A···O3 hydrogen bonds (Table 1) link adjacent molecules into two-dimensional sheets lying parallel to the *bc* plane (Fig. 2). The crystal packing is further stabilized by weak intermolecular π–π interactions [*Cg*1···*Cg*2 = 3.5812 (8) Å; symmetry code: x-1/2, -y+3/2, -z+1] involving the 1,2,3-oxadiazole and phenyl rings.

### Experimental

*N*-Nitroso-*p*-methoxyanilinoacetic acid (0.01 mol) was heated with acetic acid anhydride (0.5 mol) on a water bath for 2–3 h. The reaction mixture was kept aside at room temperature for overnight. It was then poured into ice-cold water. The obtained solid was dried and crystallized from benzene. Single crystals suitable for X-ray analysis were obtained from ethanol by slow evaporation.

### Refinement

All H atoms were located from difference Fourier map and allowed to refine freely with range of C—H = 0.95 (2) – 1.00 (2) Å. In the absence of significant anomalous dispersion, 1266 Friedel pairs were merged in the final refinement.

### Crystal data

**Table:**

C_9_H_8_N_2_O_3_	*F*(000) = 400
*M**_r_* = 192.17	*D*_x_ = 1.524 Mg m^−^^3^
Orthorhombic, *P*2_1_2_1_2_1_	Mo *K*α radiation, λ = 0.71073 Å
Hall symbol: P 2ac 2ab	Cell parameters from 2851 reflections
*a* = 7.0505 (2) Å	θ = 3.4–32.4°
*b* = 9.8220 (3) Å	µ = 0.12 mm^−^^1^
*c* = 12.0934 (3) Å	*T* = 100 K
*V* = 837.47 (4) Å^3^	Needle, yellow
*Z* = 4	0.56 × 0.15 × 0.14 mm

### Data collection

**Table:**

Bruker SMART APEXII CCD area-detector diffractometer	1760 independent reflections
Radiation source: fine-focus sealed tube	1577 reflections with *I* > 2σ(*I*)
graphite	*R*_int_ = 0.031
φ and ω scans	θ_max_ = 32.7°, θ_min_ = 2.7°
Absorption correction: multi-scan (*SADABS*; Bruker, 2009)	*h* = −10→10
*T*_min_ = 0.938, *T*_max_ = 0.984	*k* = −14→14
7384 measured reflections	*l* = −18→18

### Refinement

**Table:**

Refinement on *F*^2^	Primary atom site location: structure-invariant direct methods
Least-squares matrix: full	Secondary atom site location: difference Fourier map
*R*[*F*^2^ > 2σ(*F*^2^)] = 0.037	Hydrogen site location: inferred from neighbouring sites
*wR*(*F*^2^) = 0.100	All H-atom parameters refined
*S* = 1.06	*w* = 1/[σ^2^(*F*_o_^2^) + (0.0608*P*)^2^ + 0.0718*P*] where *P* = (*F*_o_^2^ + 2*F*_c_^2^)/3
1760 reflections	(Δ/σ)_max_ < 0.001
159 parameters	Δρ_max_ = 0.32 e Å^−^^3^
0 restraints	Δρ_min_ = −0.26 e Å^−^^3^

### Special details

**Table:**

Experimental. The crystal was placed in the cold stream of an Oxford Cryosystems Cobra open-flow nitrogen cryostat (Cosier & Glazer, 1986) operating at 100.0 (1)K.
Geometry. All esds (except the esd in the dihedral angle between two l.s. planes) are estimated using the full covariance matrix. The cell esds are taken into account individually in the estimation of esds in distances, angles and torsion angles; correlations between esds in cell parameters are only used when they are defined by crystal symmetry. An approximate (isotropic) treatment of cell esds is used for estimating esds involving l.s. planes.
Refinement. Refinement of F^2^ against ALL reflections. The weighted R-factor wR and goodness of fit S are based on F^2^, conventional R-factors R are based on F, with F set to zero for negative F^2^. The threshold expression of F^2^ > 2sigma(F^2^) is used only for calculating R-factors(gt) etc. and is not relevant to the choice of reflections for refinement. R-factors based on F^2^ are statistically about twice as large as those based on F, and R- factors based on ALL data will be even larger.

### Fractional atomic coordinates and isotropic or equivalent isotropic displacement parameters (Å^2^)

**Table:**

	*x*	*y*	*z*	*U*_iso_*/*U*_eq_	
O1	0.08268 (18)	0.95246 (11)	0.72517 (9)	0.0204 (2)	
O2	0.15734 (19)	0.83477 (13)	0.88211 (9)	0.0252 (3)	
O3	0.12932 (18)	0.57288 (10)	0.18145 (8)	0.0199 (2)	
N1	0.0695 (2)	0.92968 (13)	0.61277 (11)	0.0199 (3)	
N2	0.11891 (19)	0.80122 (12)	0.60181 (10)	0.0152 (2)	
C1	0.1632 (2)	0.82919 (14)	0.40336 (11)	0.0157 (2)	
C2	0.1665 (2)	0.77417 (15)	0.29712 (11)	0.0158 (3)	
C3	0.1284 (2)	0.63583 (15)	0.28173 (12)	0.0160 (3)	
C4	0.0868 (2)	0.55302 (15)	0.37253 (13)	0.0179 (3)	
C5	0.0829 (2)	0.60641 (15)	0.47811 (12)	0.0165 (3)	
C6	0.1211 (2)	0.74504 (16)	0.49222 (12)	0.0145 (2)	
C7	0.1622 (2)	0.73661 (15)	0.69641 (12)	0.0173 (3)	
C8	0.1394 (2)	0.83234 (16)	0.78201 (12)	0.0186 (3)	
C9	0.1403 (3)	0.65840 (16)	0.08473 (12)	0.0205 (3)	
H1A	0.192 (3)	0.928 (2)	0.4147 (15)	0.019 (5)*	
H2A	0.194 (3)	0.831 (2)	0.2340 (16)	0.024 (5)*	
H4A	0.070 (3)	0.457 (2)	0.3623 (16)	0.025 (5)*	
H5A	0.047 (3)	0.5524 (19)	0.5411 (15)	0.015 (5)*	
H7A	0.203 (3)	0.641 (2)	0.6995 (17)	0.026 (5)*	
H9A	0.124 (3)	0.603 (2)	0.0190 (16)	0.020 (5)*	
H9B	0.040 (3)	0.723 (2)	0.0852 (16)	0.023 (5)*	
H9C	0.262 (4)	0.708 (3)	0.0800 (19)	0.041 (7)*	

### Atomic displacement parameters (Å^2^)

**Table:**

	*U*^11^	*U*^22^	*U*^33^	*U*^12^	*U*^13^	*U*^23^
O1	0.0255 (5)	0.0180 (5)	0.0179 (5)	0.0014 (5)	0.0003 (5)	−0.0028 (4)
O2	0.0316 (6)	0.0267 (5)	0.0172 (5)	−0.0033 (6)	−0.0014 (5)	−0.0011 (4)
O3	0.0290 (6)	0.0155 (5)	0.0151 (5)	0.0016 (4)	−0.0015 (4)	−0.0013 (3)
N1	0.0258 (6)	0.0151 (5)	0.0188 (6)	0.0035 (5)	−0.0003 (5)	−0.0016 (4)
N2	0.0152 (5)	0.0141 (5)	0.0163 (5)	−0.0003 (4)	−0.0002 (4)	0.0006 (4)
C1	0.0168 (6)	0.0136 (5)	0.0167 (6)	−0.0008 (5)	−0.0016 (5)	0.0004 (4)
C2	0.0168 (6)	0.0136 (6)	0.0169 (6)	−0.0011 (5)	−0.0010 (5)	0.0016 (4)
C3	0.0154 (6)	0.0158 (6)	0.0169 (6)	0.0007 (5)	−0.0016 (5)	−0.0013 (4)
C4	0.0213 (7)	0.0130 (5)	0.0195 (6)	−0.0017 (5)	0.0001 (5)	0.0000 (5)
C5	0.0166 (6)	0.0138 (5)	0.0191 (6)	−0.0013 (5)	0.0004 (6)	0.0010 (5)
C6	0.0138 (6)	0.0142 (5)	0.0154 (5)	0.0001 (5)	0.0004 (5)	−0.0003 (4)
C7	0.0190 (6)	0.0167 (6)	0.0164 (6)	−0.0002 (5)	−0.0007 (5)	0.0016 (5)
C8	0.0176 (6)	0.0188 (6)	0.0192 (6)	−0.0014 (6)	−0.0003 (6)	0.0006 (5)
C9	0.0256 (7)	0.0200 (6)	0.0159 (6)	0.0019 (6)	−0.0014 (6)	−0.0002 (5)

### Geometric parameters (Å, °)

**Table:**

O1—N1	1.3807 (16)	C2—H2A	0.97 (2)
O1—C8	1.4227 (19)	C3—C4	1.398 (2)
O2—C8	1.2174 (18)	C4—C5	1.381 (2)
O3—C3	1.3613 (17)	C4—H4A	0.95 (2)
O3—C9	1.4421 (18)	C5—C6	1.398 (2)
N1—N2	1.3158 (16)	C5—H5A	0.961 (19)
N2—C7	1.3434 (17)	C7—C8	1.408 (2)
N2—C6	1.4356 (18)	C7—H7A	0.98 (2)
C1—C6	1.388 (2)	C9—H9A	0.97 (2)
C1—C2	1.3940 (19)	C9—H9B	0.95 (2)
C1—H1A	1.00 (2)	C9—H9C	0.99 (3)
C2—C3	1.3976 (19)		
			
N1—O1—C8	111.11 (12)	C4—C5—C6	118.61 (13)
C3—O3—C9	117.27 (11)	C4—C5—H5A	121.9 (11)
N2—N1—O1	103.69 (12)	C6—C5—H5A	119.4 (11)
N1—N2—C7	115.30 (12)	C1—C6—C5	121.77 (14)
N1—N2—C6	117.68 (12)	C1—C6—N2	119.22 (13)
C7—N2—C6	127.02 (12)	C5—C6—N2	119.00 (13)
C6—C1—C2	119.11 (13)	N2—C7—C8	106.54 (13)
C6—C1—H1A	121.0 (11)	N2—C7—H7A	123.4 (12)
C2—C1—H1A	119.9 (11)	C8—C7—H7A	130.1 (12)
C1—C2—C3	119.76 (13)	O2—C8—C7	137.09 (16)
C1—C2—H2A	120.5 (13)	O2—C8—O1	119.56 (14)
C3—C2—H2A	119.8 (13)	C7—C8—O1	103.34 (12)
O3—C3—C4	115.89 (13)	O3—C9—H9A	109.4 (12)
O3—C3—C2	124.01 (13)	O3—C9—H9B	110.2 (12)
C4—C3—C2	120.10 (13)	H9A—C9—H9B	107.0 (17)
C5—C4—C3	120.65 (13)	O3—C9—H9C	112.3 (15)
C5—C4—H4A	119.4 (12)	H9A—C9—H9C	109.3 (18)
C3—C4—H4A	119.8 (12)	H9B—C9—H9C	108.5 (19)
			
C8—O1—N1—N2	−0.84 (16)	C4—C5—C6—C1	−0.2 (2)
O1—N1—N2—C7	0.46 (17)	C4—C5—C6—N2	−179.47 (14)
O1—N1—N2—C6	−179.58 (12)	N1—N2—C6—C1	30.90 (19)
C6—C1—C2—C3	−0.2 (2)	C7—N2—C6—C1	−149.14 (15)
C9—O3—C3—C4	169.99 (14)	N1—N2—C6—C5	−149.86 (15)
C9—O3—C3—C2	−10.3 (2)	C7—N2—C6—C5	30.1 (2)
C1—C2—C3—O3	−179.64 (13)	N1—N2—C7—C8	0.09 (18)
C1—C2—C3—C4	0.0 (2)	C6—N2—C7—C8	−179.87 (13)
O3—C3—C4—C5	179.72 (14)	N2—C7—C8—O2	−179.70 (19)
C2—C3—C4—C5	0.0 (2)	N2—C7—C8—O1	−0.58 (16)
C3—C4—C5—C6	0.1 (2)	N1—O1—C8—O2	−179.79 (14)
C2—C1—C6—C5	0.3 (2)	N1—O1—C8—C7	0.89 (17)
C2—C1—C6—N2	179.53 (13)		

### Hydrogen-bond geometry (Å, °)

**Table:**

*D*—H···*A*	*D*—H	H···*A*	*D*···*A*	*D*—H···*A*
C1—H1A···O2^i^	1.00 (2)	2.59 (2)	3.5441 (19)	159.1 (15)
C7—H7A···O3^ii^	0.98 (2)	2.42 (2)	3.3814 (18)	165.7 (17)

Symmetry codes: (i) −*x*+1/2, −*y*+2, *z*−1/2; (ii) −*x*+1/2, −*y*+1, *z*+1/2.

## References

[bb1] Bruker (2009). *APEX2*, *SAINT* and *SADABS* Bruker AXS Inc., Madison, Wisconsin, USA.

[bb2] Cosier, J. & Glazer, A. M. (1986). *J. Appl. Cryst.* **19**, 105–107.

[bb3] Goh, J. H., Fun, H.-K., Nithinchandra, & Kalluraya, B. (2010*a*). *Acta Cryst.* E**66**, o1225–o1226. 10.1107/S1600536810015205 PMC2979081 21579251

[bb4] Goh, J. H., Fun, H.-K., Nithinchandra, & Kalluraya, B. (2010*b*). *Acta Cryst.* E**66**, o1251–o1252. 10.1107/S1600536810015667 PMC2979459 21579356

[bb5] Goh, J. H., Fun, H.-K., Nithinchandra, & Kalluraya, B. (2010*c*). *Acta Cryst.* E**66**, o1303. 10.1107/S1600536810016417 PMC2979510 21579399

[bb6] Hegde, J. C., Girisha, K. S., Adhikari, A. & Kalluraya, B. (2008). *Eur. J. Med. Chem.* **43**, 2831–2834. 10.1016/j.ejmech.2008.02.003 18387710

[bb7] Kalluraya, B. & Rahiman, A. M. (1997). *Pol. J. Chem.* **71**, 1049–1052.

[bb8] Kalluraya, B., Rahiman, M. A. & Banji, D. (2002). *Indian J. Chem. Sect. B*, **41**, 1712–1717.

[bb9] Rai, N. S., Kalluraya, B., Lingappa, B., Shenoy, S. & Puranic, V. G. (2008). *Eur. J. Med. Chem.* **43**, 1715–1720. 10.1016/j.ejmech.2007.08.002 17923171

[bb10] Sheldrick, G. M. (2008). *Acta Cryst.* A**64**, 112–122. 10.1107/S0108767307043930 18156677

[bb11] Spek, A. L. (2009). *Acta Cryst.* D**65**, 148–155. 10.1107/S090744490804362X PMC2631630 19171970

